# Visual Tracking Using Sparse Coding and Earth Mover's Distance

**DOI:** 10.3389/frobt.2018.00095

**Published:** 2018-08-22

**Authors:** Gang Yao, Ashwin Dani

**Affiliations:** Department of Electrical and Computer Engineering, University of Connecticut Storrs, CT, United States

**Keywords:** visual tracking, earth mover's distance, sparse coding, gyro-aided tracking, max-alignment pooling, template update

## Abstract

An efficient iterative Earth Mover's Distance (iEMD) algorithm for visual tracking is proposed in this paper. The Earth Mover's Distance (EMD) is used as the similarity measure to search for the optimal template candidates in feature-spatial space in a video sequence. The local sparse representation is used as the appearance model for the iEMD tracker. The maximum-alignment-pooling method is used for constructing a sparse coding histogram which reduces the computational complexity of the EMD optimization. The template update algorithm based on the EMD is also presented. When the camera is mounted on a moving robot, e.g., a flying quadcopter, the camera could experience a sudden and rapid motion leading to large inter-frame movements. To ensure that the tracking algorithm converges, a gyro-aided extension of the iEMD tracker is presented, where synchronized gyroscope information is utilized to compensate for the rotation of the camera. The iEMD algorithm's performance is evaluated using eight publicly available videos from the CVPR 2013 dataset. The performance of the iEMD algorithm is compared with eight state-of-the-art tracking algorithms based on relative percentage overlap. Experimental results show that the iEMD algorithm performs robustly in the presence of illumination variation and deformation. The robustness of this algorithm for large inter-frame displacements is also illustrated.

## 1. Introduction

Visual tracking is an important problem for new robotics applications. The information generated from the sequence of images by the tracking algorithm can be utilized by vehicle navigation, human-robot interaction, and motion-based recognition algorithms (Dani et al., 2013; Ravichandar and Dani, 2015; Chwa et al., 2016). Visual tracking algorithms provide important information for visual simultaneous localization and mapping (SLAM), structure from motion (SfM), and vision-based control (Marchand and Chaumette, 2005; Marchand et al., 2005; Comport et al., 2006; Davison et al., 2007; Dani et al., 2012; Yang et al., 2015).

Image-based tracking algorithms are categorized as point tracking, kernel tracking, or silhouette tracking (Yilmaz et al., 2006). Distinguishing features, such as color, shape, and region are selected to identify objects for visual tracking. Modeling the object adapts to the slowly changing appearance is challenging, due to the illumination variants, object deformation, occlusion, motion blur, or background clutters. Supervised or unsupervised online learning algorithms are often used to robustly find and update the distinguishing features of the object, such as using variance ratios of the feature value's log likelihood (Collins et al., 2005), the online Ada-boost feature selection method (Grabner and Bischof, 2006), and incremental learning (Ross et al., 2008).

Approaches in visual tracking could be generally classified into two groups, either generative methods or discriminative methods. For generative methods, the tracked object is modeled based on the selected features, such as the color histogram, sparse coding representation, or kernels. Then, correspondence or similarity measurement between the target and the candidate across frames is constructed. Similarity measurements are derived through several methods, such as the Normalized Cross Correlation (NCC) (Bolme et al., 2010; Zhu et al., 2016), the Earth Mover's Distance (EMD) (Zhao et al., 2010; Karavasilis et al., 2011; Oron et al., 2012; Tahri et al., 2016), the Bhattacharyya Coefficient (BC) (Comaniciu et al., 2003) and point-to-set distance metric (Wang et al., 2015, 2016). Location of the candidate object in the consecutive frames is estimated by using the Kalman filter, particle filter or gradient descent method. Discriminative methods regard tracking as a classification problem and build a classifier or ensemble of classifiers to distinguish the object from the background. Representative classification tracking algorithms are the structured Support Vector Machine (SVM) (Hare et al., 2011), Convolutional Neural Nets (Li et al., 2016). Ensemble based algorithms such as ensemble tracking (Avidan, 2007), multiple instance learning (MIL) (Babenko et al., 2011), and online boosting tracker (Grabner and Bischof, 2006).

In order to robustly track moving objects in challenging situations, many tracking frameworks are proposed. Tracking algorithms with Bayesian filtering are developed to track moving objects. These algorithms can handle complete occlusion (Zivkovic et al., 2009). The non-adaptive methods, usually only model the object from the first frame. Although less error prone to occlusions and drift, they are hard to track the object undergoing appearance variations. However, adaptive meth ods are usually prone to drift because they rely on self updates of an online learning method. In order to deal with this problem, combining adaptive methods with the complementary tracking approaches leads to more stable results. For example, parallel robust online simple tracking (PROST) framework combines three different trackers (Santner et al., 2010): tracking-learning-detection (TLD) framework uses P-N experts to make the decision on the location of the moving object, based on the results from the Median-Flow tracker and detectors (Kalal et al., 2012), and online adaptive hidden Markov model for multi-tracker fusion (Vojir et al., 2016).

An EMD-based tracker using color histogram [iEMD(CH)] as an appearance model and its fusion with gyroscope information is presented in our prior related work (Yao et al., 2016; Yao and Dani, 2017). However, color histogram model is not robust to appearance changes. Also, the template update algorithm is not used in our prior related work. The sparse coding appearance model is based on a dictionary of templates consisting of the appearance variations of the target. The sparse coding appearance model has been used in literature and has shown robust performance in various tracking algorithms (Zhang et al., 2013). In this paper, we develop a generative tracking method using sparse coding appearance model along with EMD as a similarity measure. An adaptive template update algorithm is also developed to update the apprearance model during tracking to handle the appearance variations. Gyroscope information is used to aid the initialization of the EMD optimization. Specifically, the contributions of the paper are

- The maximum-alignment-pooling method for local sparse coding is used to build a histogram of appearance model. A template update algorithm is used to adaptively change the appearance model by an exponential rule based on EMD measure. An iEMD tracking algorithm is developed based on this local sparse coding representation of the appearance model. It is shown using videos from publicly available benchmark datasets that the iEMD tracker shows good performance in terms of percentage overlap compared to the state-of-the-art trackers available in literature.
- Gyro-measurements are used to compensate for the pan, tilt, and roll of the camera. Then the iEMD visual tracking algorithm is used to track the target after compensating for the movement of the camera. By this method, the convergence of the algorithm is ensured, thus providing a more robust tracker which is more capable of real-world tracking tasks.

The paper is organized as follows. Related work on the computation of the EMD and its application for visual tracking is illustrated in section 2. In section 3, the iEMD algorithm for visual tracking is developed. In section 4, the target is modeled as the sparse coding histogram. For the sparse coding histogram, the maximum-alignment-pooling method is proposed to represent the local image patches. In section 5, two extensions of the iEMD algorithm that includes the template update method, and the method of using the gyroscope data for ego-motion compensation are discussed. In section 6, the iEMD tracker is validated on eight publicly available datasets, and the comparisons with eight state-of-the-art trackers are shown. Experimental results using the gyro-aided iEMD algorithm are compared with tracking results without gyroscope information. The conclusions are given in section 7.

## 2. Related work

Sparse coding has been successfully applied to model the target in visual tracking (Zhang et al., 2013). In sparse coding for visual tracking, the largest sum of the sparse coefficients or the smallest reconstruction error is used as the metric to find the target from the candidate templates using particle filter (Mei and Ling, 2009; Jia et al., 2016). The sparse coding process is usually the *L*_1_ norm minimization problem, which makes the sparse representation and dictionary learning computationally expensive. To reduce the computational complexity, the sparse representation as the appearance model is combined with the Mean-shift (Liu et al., 2011) or Mean-transform method (Zhang and Hong Wong, 2014). After a small number of iterations by these methods, the maximum value of the Bhattacharyya coefficient corresponding to the best candidate is obtained.

In real-world tracking applications, variations in appearance are a common phenomenon caused by illumination changes, moderate pose changes or partial occlusions. The Earth Mover's Distance (EMD) as a similarity measure, also known as 1-Wasserstein distance (Guerriero et al., 2010; Baum et al., 2015), is robust to these situations (Rubner et al., 2000). However, the major problem with the EMD is its computational complexity. Several algorithms for the efficient computation of the EMD are proposed. For example, the EMD-*L*_1_ algorithm is used for histogram comparison (Ling and Okada, 2007) and the EMDs are computed with the thresholded ground distances (Pele and Werman, 2009). In the context of visual tracking, although the EMD has the merit of being robust to moderate appearance variations, the efficiency of the computation is still a problem. Since solving the EMD is a transportation problem—a linear programming problem (Rubner et al., 2000), the direct differential method cannot be used. There are some efforts to employ the EMD for object tracking. The Differential Earth Mover's Distance (DEMD) algorithm (Zhao et al., 2010) is first proposed for visual tracking, which adopts the sensitivity analysis to approximate the derivative of the EMD. However, the selection of the basic variables and the process of identifying and deleting the redundant constraints still affect the efficiency of the algorithm (Zhao et al., 2010). The DEMD algorithm combined with the Gaussian Mixture Model (GMM), which has fewer parameters for EMD optimization, is proposed in Karavasilis et al. (2011). The EMD as the similarity measure combined with the particle filter for visual tracking is proposed in Oron et al. (2012). In this paper, the sparse coding is used along with EMD similarity measure for the visual tracking. To the best of our knowledge, this is the first work that combines sparse coding representation with the EMD similarity measure for visual tracking.

The success of the gradient descent based tracking algorithm depends on the assumption that the object motion is smooth and contains only small displacements (Yilmaz et al., 2006). However, in practice, this assumption is always violated due to the abrupt rotation and shaking movement of the camera mounted on a mobile robot, such as a flying quadcopter. Efforts have been made to combine the gyroscope data with tracking algorithms, such as the Kanade-Lucas-Tomasi (KLT) tracker or the MI tracker (Hwangbo et al., 2011; Park et al., 2013; Ravichandar and Dani, 2014). In our paper, to robustly track a static object using a moving camera, gyroscope data are directly utilized to estimate the initial location of the static object. When both the camera and the object being tracked are in motion, the gyroscope sensor data are utilized to compensate for the rotation of the camera, because rotation has a greater impact on the positional changes compared with the translation in video frames. Then, the iEMD tracking algorithm is applied to track the moving object. The robustness of the tracking algorithm is improved due to the compensation of the camera's ego-motion. Therefore, our method makes the EMD tracker more robust to this situation.

## 3. Iterative EMD tracking algorithm

In the context of visual tracking, first a feature space is chosen to characterize the object, then, the target model and the candidate model are built in the feature-spatial space. The probability density functions (histograms) representing the target model and the candidate model are (Comaniciu et al., 2003)

$$\text{target model}:\hat{p}={\left\{{\hat{p}}_{u}\right\}}_{u=1,\ldots ,N_{T}}\text{ and }\sum_{1}^{N_{T}}{\hat{p}}_{u}=1$$

$$\text{candidate model}:\hat{q}(y)={\left\{{\hat{q}}_{v}(y)\right\}}_{u=1,\ldots ,N_{C}}\text{and }\sum_{1}^{N_{C}}{\hat{q}}_{u}(y)=1,$$

where ${\hat{p}}_{u}$ is the weight of the *u*th bin of the target model $\hat{\text{p}}$, assuming the center of the template target is at (0, 0), ${\hat{q}}_{v}$ is the weight of the *v*th bin of the candidate model $\hat{\text{q}}\left(\text{y}\right)$, assuming the center of the template candidate is at **y**, *N*_*T*_ and *N*_*C*_ are the numbers of the bins.

Based on the target model and the candidate model, the dissimilarity function is denoted as $f\left(\hat{\text{p}},\hat{\text{q}}\left(\text{y}\right)\right)$. The optimization problem for tracking is to estimate the optimal displacement $\hat{\text{y}}$ which gives the smallest value of $f\left(\hat{\text{p}},\hat{\text{q}}\left(\text{y}\right)\right)$. Thus, the optimization problem is formulated as

(1) $$\begin{array}{l} \hat{\text{y}}=\arg\;\underset{\text{y}}{\min}\;f\left(\hat{\text{p}},\hat{\text{q}}\left(\text{y}\right)\right) \end{array}$$

In (1), the center of the template target is assumed to be positioned at (0, 0), and the center of the template candidate is at **y**. The goal is to find the candidate model located at $\hat{\text{y}}$ that gives the smallest value of the dissimilarity function $f\left(\hat{\text{p}},\hat{\text{q}}\left(\text{y}\right)\right)$. The differential tracking approaches are usually applied to solve this optimization problem, with the assumption that the displacement of the target between two consecutive frames is very small.

The optimization problem in (1) is solved using the iEMD algorithm as described in the following sub-sections. The iEMD algorithm iterates between finding the smallest EMD between template target and the template candidate based on the current position **y**_*k*_ by the transportation-simplex method (see section 3.2 for details) and finding the best position **y**_*k*+1_ leading to the smallest EMD by gradient method (see section 3.3 for details).

### 3.1. EMD as a similarity measure

**Figure 1:**
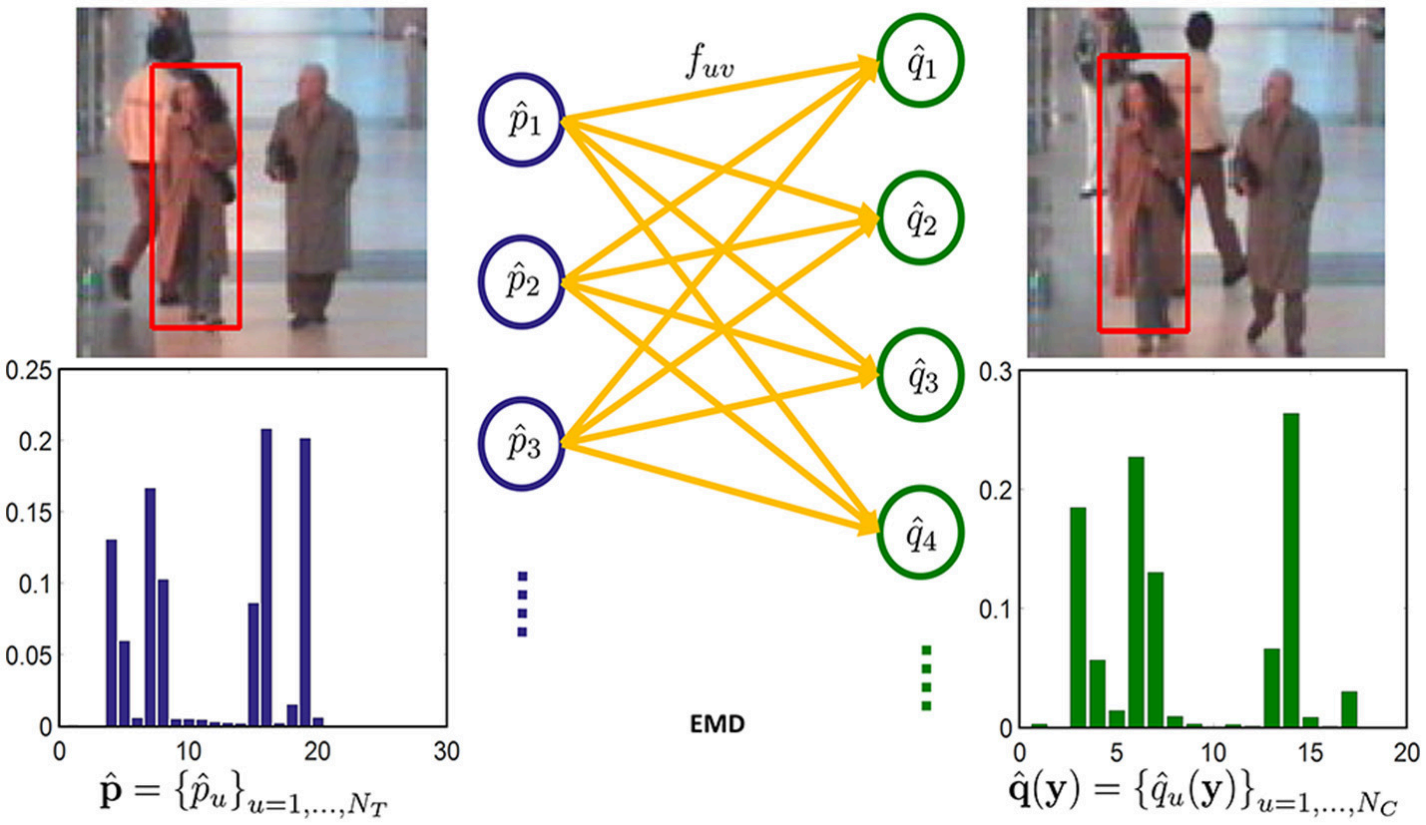
EMD comparison of the two templates (No permission is required from the copyright holders and/or the depicted individuals for the use of these images. The original images are obtained from the EC Funded CAVIAR project/IST 2001 37540, found at http://homepages.inf.ed.ac.uk/rbf/CAVIAR/).

In this section, the EMD between the target model $\hat{\text{p}}$ and the candidate model $\hat{\text{q}}\left(\text{y}\right)$ is used as the similarity measure. Solving the EMD is a transportation problem—a linear programming problem as shown in Figure 1. Intuitively, given the target model and the candidate model, each bin of both models are cross compared. The costs between the bins from two different models are predefined. Then the EMD is considered as the smallest overall cost of sending the weights of one bin from the target model to another bin of the candidate model. The EMD is defined as (Rubner et al., 2000)

(2) $$\begin{array}{l} D^{⋆}\left(f_{uv}\left(\hat{\text{p}},\hat{\text{q}}\left(\text{y}\right)\right)\right)≜\underset{f_{uv}}{\min}\left(\overset{N_{T}}{\underset{u=1}{\sum }}\overset{N_{C}}{\underset{v=1}{\sum }}d_{uv}f_{uv}\left(\hat{\text{p}},\hat{\text{q}}\left(\text{y}\right)\right)\right) \end{array}$$

subject to

(3) $$\begin{array}{l} \overset{N_{T}}{\underset{u=1}{\sum }}f_{uv}\left(\hat{\text{p}},\hat{\text{q}}\left(\text{y}\right)\right)=w_{C,v},1\leq v\leq N_{C} \end{array}$$

(4) $$\begin{array}{l} \overset{N_{C}}{\underset{v=1}{\sum }}f_{uv}\left(\hat{\text{p}},\hat{\text{q}}\left(\text{y}\right)\right)=w_{T,u},1\leq u\leq N_{T} \end{array}$$

(5) $$\begin{array}{l} \overset{N_{T}}{\underset{u=1}{\sum }}\overset{N_{C}}{\underset{v=1}{\sum }}f_{uv}\left(\hat{\text{p}},\hat{\text{q}}\left(\text{y}\right)\right)=1 \end{array}$$

(6) $$\begin{array}{l} f_{uv}\left(\hat{\text{p}},\hat{\text{q}}\left(\text{y}\right)\right)\geq 0,1\leq u\leq N_{T},1\leq v\leq N_{C} \end{array}$$

where *D*^⋆^ is the optimal solution to this transportation problem, $f_{uv}\left(\hat{\text{p}},\hat{\text{q}}\left(\text{y}\right)\right)$ is the flow (weight) from the *u*th bin of $\hat{\text{p}}$ to the *v*th bin of $\hat{\text{q}}\left(\text{y}\right)$, *d*_*uv*_ is the ground distance (cost) between the *u*th and the *v*th bins, the subscript *T* denotes the object target and *C* is the object candidate, *w*_*T, u*_ is the weight from the *u*th bin of $\hat{\text{p}}$, and *w*_*C, v*_ is the weight from the *v*th bin of $\hat{\text{q}}\left(\text{y}\right)$.

### 3.2. EMD as a function of weights

Writing the above equation set (2–6) in a matrix form as

(7) $$\begin{array}{l} D^{⋆}=\underset{f}{\text{min}}\;d^{T}f\\ \text{s}.\text{t}.Hf=w;\;f>0 \end{array}$$

where the $\text{d}={\left[d_{11},\cdots d_{1N_{C}},\cdots d_{N_{T}1},\cdots d_{N_{T}N_{C}}\right]}^{T}\in {\mathbb{R}}^{N_{T}N_{C}}$ is the ground distance vector, $f\;=\;{\left[f_{11},\cdots f_{1,N_{C}},\cdots f_{N_{T}1,}\cdots f_{N_{T}N_{C}}\right]}^{T}\in {\mathbb{R}}^{N_{T}N_{C}}$is the flow vector, $w={\left[w_{N_{C}}^{T},w_{N_{T}}^{T}\right]}^{T}\in {\mathbb{R}}^{N_{T}+N_{C}}$ is the weight vector consisting of the weight vectors $w_{N_{C}}\in {\mathbb{R}}^{N_{C}}$ from $\hat{\text{q}}\left(\text{y}\right)$ and $w_{N_{T}}\in {\mathbb{R}}^{N_{T}}$ from $\hat{\text{p}}$, and $H\in {\mathbb{R}}^{(N_{T}+N_{C})\times N_{T}N_{C}}$ is the matrix which consists of 0 and 1*s*.

In order to relate the EMD with the weight vector, the above primal problem in (7) is restated in its dual form as (Dantzig and Thapa, 2006)

(8) $$\begin{array}{l} D^{⋆}=\underset{\pi }{\text{max }}\;w^{T}\pi \\ \text{s}.\text{t}.\;H^{T}\pi \leq d \end{array}$$

where $\pi \in {\mathbb{R}}^{N_{T}+N_{C}}$ is a vector of variables to be optimized in the dual problem. By solving this dual problem in (8), the optimal solution *D*^⋆^ is calculated and directly represented as the linear equation of weights. However, considering the computation efficiency, the optimal solution (EMD) is first calculated from the primal problem in (7) using the transportation-simplex method, and then the EMD is represented as the function of the weights by the matrix transformation.

Using the transportation-simplex method (Rubner et al., 2000), the optimal solution to the EMD problem in (7) is calculated. The transportation-simplex method is a streamlined simplex algorithm, which is built on the special structure of the transportation problem. In order to reduce the number of iterations of the transportation-simplex method, the Russell's method is used to compute the initial basic feasible solution (Rubner et al., 2000; Ling and Okada, 2007).

The computation of the EMD is a transportation problem, which has exactly *N*_*T*_ + *N*_*C*_−1 basic variables $f_{B}\in {\mathbb{R}}^{N_{T}+N_{C}-1}$, and each constraint is a linear combination of the other *N*_*T*_ + *N*_*C*_ − 1 constraints, which could be considered as redundant and discarded (Dantzig and Thapa, 2006). Based on the optimal solution to the linear programming problem, the flow vector is separated into basic variables and non-basic variables $f={\left[f_{B}^{T},f_{NB}^{T}\right]}^{T}\in {\mathbb{R}}^{N_{T}N_{C}},$, and the ground distance vector **d** and **H** will be transformed as $d={\left[d_{B}^{T},d_{NB}^{T}\right]}^{T}\in {\mathbb{R}}^{N_{T}N_{C}}$ and $H={\left[H_{B},H_{NB}\right]}^{T}\in {\mathbb{R}}^{(N_{T}+N_{C})\times N_{T}N_{C}},$, where $d_{B}\in {\mathbb{R}}^{N_{T}+N_{C}-1}$, and $H_{B}\in {\mathbb{R}}^{\left(N_{T}+N_{C})\times (N_{T}+N_{C}-1\right)}.$. In order to derive the EMD as a function of the weights of the candidate model, the matrix transformation is performed. First, the last row of the constraint matrices (7) is deleted which is considered as the redundant constraint and then the matrices **H***_B_*, **H**, and **w** are formulated as $H_{B}^{*}\;\in {\mathbb{R}}^{\left(N_{T}+N_{C}-1)\times (N_{T}+N_{C}-1\right)}$, $H^{*}={\left[H_{B}^{*},H_{NB}^{*}\right]}^{T}\in {\mathbb{R}}^{(N_{T}+N_{C}-1)\times N_{T}N_{C}}$ and $w^{*}={\left[w_{N_{C}}^{T},w_{N_{T}-1}^{T}\right]}^{T}\in {\mathbb{R}}^{N_{T}+N_{C}-1}.$.

The problem in (7) is reformulated based on the optimal solution as

(9) $$\begin{array}{l} D^{⋆}-{d_{B}}^{T}f_{B}-{d_{NB}}^{T}f_{NB}\;=0 \end{array}$$

(10) $$\begin{array}{l} H_{B}^{*}f_{B}+H_{NB}^{*}f_{NB}\;=w^{*} \end{array}$$

Left multiplying (10) with $H_{B}^{*-1}$ yields

(11) $$\begin{array}{l} f_{B}+H_{B}^{*-1}H_{NB}^{*}f_{NB}=H_{B}^{*-1}w^{*} \end{array}$$

Left multiplying (11) by ${d_{B}}^{T}$ and adding the resultant to (9) gives

(12) $$\begin{array}{l} D^{⋆}+(-{d_{NB}}^{T}+MH_{NB}^{*})f_{NB}=Mw^{*} \end{array}$$

where $M={d_{B}}^{T}H_{B}^{*-1}$ is a *N*_*C*_+*N*_*T*_−1-dimensional vector. Since **f_NB_** = **0**_*N*_*T*_*N*_*C*_−*N*_*T*_−*N*_*C*_+1_, using (12) the EMD *D*^⋆^ is given by

(13) $$\begin{array}{l} D^{⋆}=M{\left[w_{N_{C}}^{T},0\right]}_{N_{C}+N_{T}-1}^{T}+M{\left[0,w_{N_{T}-1}^{T}\right]}_{N_{C}+N_{T}-1}^{T} \end{array}$$

### 3.3. Gradient method to find the template displacement

Based on the Equation (13), the gradient method is utilized to find the displacement **y** of the target candidate as

(14) $$\begin{array}{l} \frac{\partial D^{⋆}}{\partial y}=M{\left[\frac{\partial w_{N_{C}}^{T}}{\partial y},0\right]}_{N_{C}+N_{T}-1}^{T} \end{array}$$

The optimal location $\hat{\text{y}}$ of the template candidate $\hat{\text{q}}\left(\text{y}\right)$ is found by iteratively performing: (1) calculate the smallest EMD and reformulate it as (13); (2) search for the new location of the template candidate along the direction of (14). When the EMD no longer decreases, the iteration stops. By this method, the best match of the template target and the template candidate will be found. The EMD plays three roles in this algorithm: (1) it provides a metric of the matching between the template target and the template candidate; (2) it assigns more weights to the best matches between the histogram bins and assigns smaller weights or no weights to unmatched bins by linear optimization; (3) matched bins are used for finding the location of the template candidate, and the gradient vector of the EMD for searching the optimal displacement is calculated.

The pseudo-code for the iEMD tracking algorithm is given in Algorithm 1.

**Algorithm 1:**
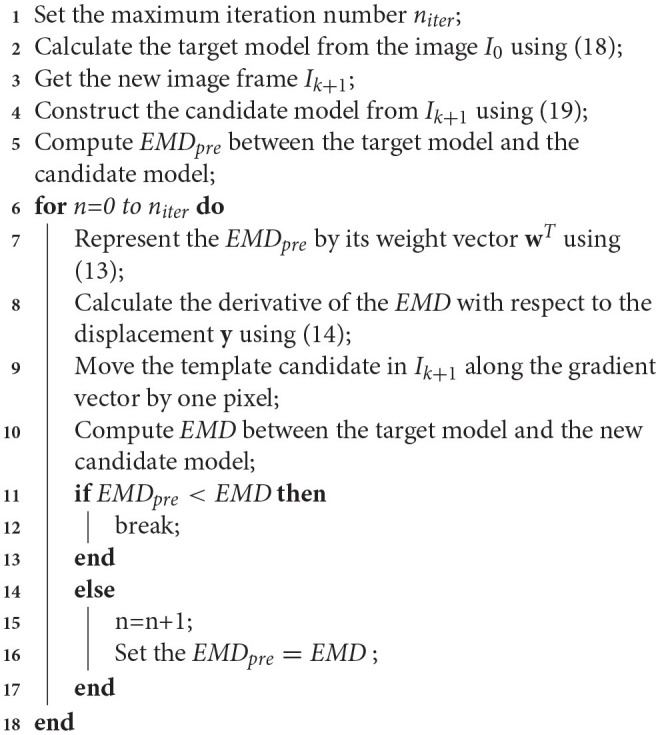
iEMD tracking algorithm.

## 4. Target modeling based on histograms of sparse codes

Sparse codes histogram (SCH) has been widely used as feature descriptors in many fields (Zhang et al., 2013). Given the image set of the first *L* image templates from a video, a set of *K* overlapped local image patches are sampled by a sliding window of size *m* × *n* from each template to build a dictionary **Φ** ∈ ℝ^(*mn*) × (*LK*)^. Each column of **Φ** is a basis vector, which is a vectorized local image patch extracted from the set of image templates. The basis vectors are overcomplete where *mn* < *LK*. Similarly, for a given image template target *I*, a set of overlapped local image patches $\text{E}=\left\{\epsilon_{r}|\epsilon_{r}\in {\mathbb{R}}^{\left(mn\right)\times 1},r=1\cdots J\right\}$ are sampled by the same sliding window of size *m*×*n* with the step size of one pixel. Each image patch ϵ_*r*_, which represents one fixed part of the target object, can be encoded as a linear combination of a few basis vectors of the dictionary **Φ** as follows

(15) $$\begin{array}{l} \epsilon_{r}=\Phi {\text{a}}_{r}+\text{n} \end{array}$$

where ${\text{a}}_{r}\in {\mathbb{R}}^{\left(LK\right)\times 1}$ is the coefficient vector which is sparse and **n** ∈ ℝ^(*mn*) × 1^ is the noise vector. The coefficient **a**_*r*_ is computed by solving the following *L*_1_ norm minimization problem (Zhang et al., 2013; Mairal et al., 2014)

(16) $$\begin{array}{l} \underset{a_{r}}{\text{min}}{\left\|\epsilon_{r}-\Phi a_{r}\right\|}_{2}^{2}+\lambda {\left\|a_{r}\right\|}_{1}\\ \text{s}.\text{t}.\;{\left(a_{r}\right)}_{k}\geq 0,\forall k \end{array}$$

where ${\text{a}}_{r}={\left[\begin{array}{ccccccc} a_{11}, & \cdots \;, & a_{1K}, & \cdots \;, & a_{L1}, & \cdots \;, & a_{LK} \end{array}\right]}^{T}$ is the sparse coefficients of the local patch, *a*_*ij*_ corresponds to the jth patch of the *i*th image template of the dictionary, and λ is the Lagrange multiplier.

Once a solution to (16) is obtained, the maximum-alignment-pooling method is used to construct the sparse coding histograms. Combining the coefficients corresponding to the dictionary patches that have the same locations in the template using ${ā}_{j}=\overset{L}{\underset{i=1}{\sum }}a_{ij}$ (Jia et al., 2012), a new vector ${\bar{\text{a}}}_{r}={\left[\begin{array}{ccc} {ā}_{1}, & \cdots \;, & {ā}_{j} \end{array}\right]}^{T}\in {\mathbb{R}}^{K\times 1}$ is formulated. The weight of the *r*th local image patch ϵ_*r*_ in the histogram of sparse codes is computed by using ${\hat{p}}_{ru}={{\bar{\text{a}}}_{r}}_{\infty }$. The ${\hat{p}}_{ru}$ value corresponds to the *u*th image patch from ${\bar{\text{a}}}_{r}$. With *J* local image patches from the template target, the histogram is constructed as

(17) $$\begin{array}{l} \hat{\text{p}}={\left[\begin{array}{c} {\hat{p}}_{11},\cdots \;,{\hat{p}}_{ru},\cdots \;,{\hat{p}}_{JK} \end{array}\right]}^{T}\in {\mathbb{R}}^{J\times 1} \end{array}$$

In the spatial space, the Epanechnikov kernel is used to represent the template. The Epanechnikov kernel (Comaniciu et al., 2003) is an isotropic kernel with a convex profile which assigns smaller weights to pixels away from the center. Given the target histogram $\hat{\text{p}}$ in (17), the isotropic kernel is applied to generate the histograms of target weighted by the spatial locations. The weights of the histogram of the target *w*_*T, u*_ are computed using

(18) $$\begin{array}{l} w_{T,u}=\gamma \overset{J}{\underset{r=1}{\sum }}\left(1-{\frac{{\text{c}}_{r}}{h}}^{2}\right)|{\hat{p}}_{ru}| \end{array}$$

where **c**_*r*_ is the center of the *r*th image patch of the template target, *h* is template size and γ is the normalization constant. The candidate histogram $\hat{\text{q}}$ is built in the same way as $\hat{\text{p}}$. An isotropic kernel is applied to the elements of the $\hat{\text{q}}$ for generating the histogram of candidate with spatial locations. The weights of the candidate histogram *w*_*C, v*_(**x**_*i*_−**y**) are computed using

(19) $$\begin{array}{l} w_{C,v}(x_{i}-y)=\gamma \sum_{r=1}^{J}\left(1-{\left\|\frac{c_{r}-y}{h}\right\|}^{2}\right)\left|{\hat{q}}_{rv}\right| \end{array}$$

where **y** is the displacement of the *r*th image patch of the template candidate. The ground distance *d*_*uv*_ for the EMD in (2) is defined by

(20) $$\begin{array}{l} d_{uv}=\alpha {\left\|\epsilon_{u}-\epsilon_{v}\right\|}_{2}^{2}+\left(1-\alpha \right){\left\|c_{u}-c_{v}\right\|}_{2}^{2} \end{array}$$

where α ∈ (0, 1) is the weighting coefficient, $\epsilon_{u}\in {\mathbb{R}}^{\left(mn\right)\times 1}$, $\epsilon_{v}\in {\mathbb{R}}^{\left(mn\right)\times 1}$ are the vectors of the normalized pixel values of the image patch from the target and candidate templates, sampled in the same way as the image patches from the dictionary, and **c**_*u*_, **c**_*v*_ are the corresponding centers of the image patches.

## 5. Extensions of the tracking algorithm

### 5.1. Template update

In order to make the tracker robust to significant appearance variations during long video sequences, the outdated templates in the dictionary should be replaced with the recent ones. To adapt to the appearance variations of the target and alleviate the drift problem only the latest template in dictionary is replaced based on the weight ω_*i*_, which is computed by

(21) $$\begin{array}{l} \omega_{i}=\gamma_{0}^{\Delta i}\times \exp\left(-D_{k}^{*}\right) \end{array}$$

where ω_*i*_ is the weight associated with the template, γ_0_ is a constant, Δ*i* is the time elapsed since the dictionary was last updated measured in terms of image index *k* and $D_{k}^{*}$ is the EMD value corresponding to the template *I*_*k*_.

If the weight of the current template based on (21) is smaller than the weight of the latest template in the dictionary, the template is replaced with the current one. In order to avoid the errors and noises affecting the dictionary update algorithm, the reconstructed template is used to replace the one in the dictionary. Firstly, the following problem is solved in order to recompute the sparse code coefficients, **a**_*k*_,

(22) $$\begin{array}{l} \underset{a_{k}}{\text{min}}{\left\|I_{k}-\left[\begin{array}{cc} \Phi_{\text{T}} & I_{mn\times mn} \end{array}\right]a_{k}\right\|}_{2}^{2}+\lambda {\left\|a_{k}\right\|}_{1} \end{array}$$

where $\Phi_{\text{T}}\in {\mathbb{R}}^{(mn)\times K}$ is a dictionary formed using the vectorized template image with the size *m* × *n* as columns, **I**_*mn* × *mn*_ is the identity matrix, ${\text{a}}_{k}\in {\mathbb{R}}^{K+mn}$ is the vector of the sparse coding coefficients, and λ is the Lagrange multiplier (cf., Jia et al., 2012). Then the reconstructed template is calculated using $\Phi_{\text{T}}a_{k}^{*}$, where ${\text{a}}_{k}^{*}\in {\mathbb{R}}^{K}$ is computed using components of **a**_*k*_ corresponding to the dictionary. The reconstructed template is used to replace the latest template in the dictionary. The detailed steps of the update scheme are given in Algorithm 2.

**Algorithm 2:**
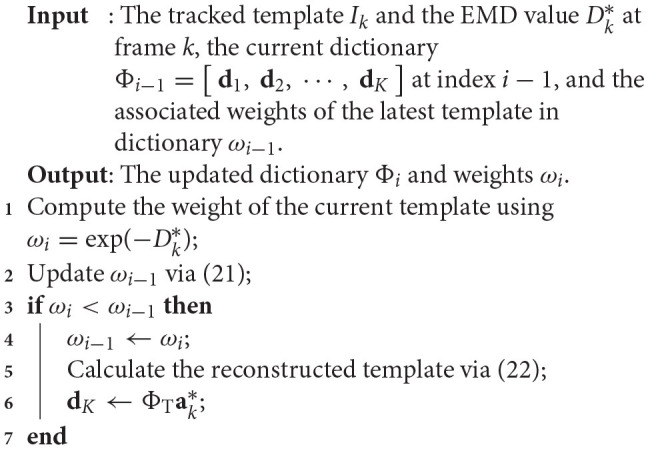
Template update procedure.

### 5.2. Gyroscope data fusion for rotation compensation

The general idea of the gyro-aided iEMD tracking algorithm is combining the image frames from the camera with the angular rate generated by the gyroscope for visual tracking. Synchronization of the camera and the gyroscope in time is required. The spatial relationship between the camera and the gyroscope must also be pre-calibrated. Then, the angular rate generated by the gyroscope is applied to compensate for the ego-motion of the camera. After the compensation of the ego-motion of the camera, the iEMD tracker is applied for tracking. In this section, details of the gyro-aided iEMD tracking algorithm are explained.

When a camera is mounted on a moving robot, the motion of the camera will cause a large displacement of the target between two consecutive frames. If the displacement is larger than the convergence region, the tracking algorithm will become susceptible to the large appearance changes and fail (Comaniciu et al., 2003; Hwangbo et al., 2011; Ravichandar and Dani, 2014). In order to improve the robustness of the tracking algorithm, the displacement caused by the camera rotation is estimated and compensated by fusing the data from the gyroscope, which is a commonly used sensor on flying robots. The rotation of the camera causes a larger displacement of the target compared with the translation movement in video-rate frames. Thus, the translation is neglected here.

The gyroscope provides the angular rate along three axes, which measure the pan, tilt, and roll of small time intervals Δ*t*. In the case of pure rotation without translation, the angular rate ω_*y*_ is obtained along three axes *x*, *y* and *z*. Let *q*(*k*), *q*(*k*+1) ∈ ℍ denote the quaternion of two frames *k* and *k*+1 during time Δ*t*, the relationship between them is given as (cf. Spong et al., 2006)

(23) $$\begin{array}{l} q\left(k+1\right)=q\left(k\right)+\frac{1}{2}\Omega \left(\omega \right)\cdot q\left(k\right)\cdot \Delta t \end{array}$$

where Ω(ω) is the skew-symmetric matrix of ω as

(24) $$\begin{array}{l} \Omega \left(\omega \right)=\left[\begin{array}{cc} 0 & -\omega^{T}\\ \omega & -{\left[\omega \right]}_{\times } \end{array}\right] \end{array}$$

After the quaternion *q*(*k*+1) = *m*+*a***i**+*b***j**+*c***k** is normalized and updated, the rotation matrix $R_{k}^{k+1}$ is calculated as

(25) $$\begin{array}{l} R_{k}^{k+1}=\left[\begin{array}{ccc} 1-2b^{2}-2c^{2} & 2ab-2cm & 2ac+2bm\\ 2ab+2cm & 1-2a^{2}-2c^{2} & 2bc-2am\\ 2ac-2bm & 2bc+2am & 1-2a^{2}-2b^{2} \end{array}\right] \end{array}$$

Thus, the estimated homography matrix between two templates is estimated by

(26) $$\begin{array}{l} H_{\text{gyro}}=KR_{k}^{k+1}K^{-1} \end{array}$$

where, *K* is the intrinsic camera calibration matrix that is accessed by calibrating the camera. The homography matrix is update d to the newest frame location $p\left(k+1\right)={\left[x_{c},y_{c},1\right]}^{T}$, where (*x*_*c*_, *y*_*c*_) is the center point of the template, based on the following equations:

(27) $$\begin{array}{l} H^{k+1}=H^{k}H_{\text{gyro}}^{-1} \end{array}$$

(28) $$\begin{array}{l} p\left(k+1\right)=H^{k+1}\cdot p\left(k\right) \end{array}$$

for the first frame, $H^{0}={\text{I}}_{3\times 3}$. This new location $p\left(k+1\right)={\left[x_{c},y_{c},1\right]}^{T}$ is then used as the initial guess of the object candidate.

The pseudo-code for gyro-aided iEMD algorithm is given in Algorithm 3.

**Algorithm 3:**
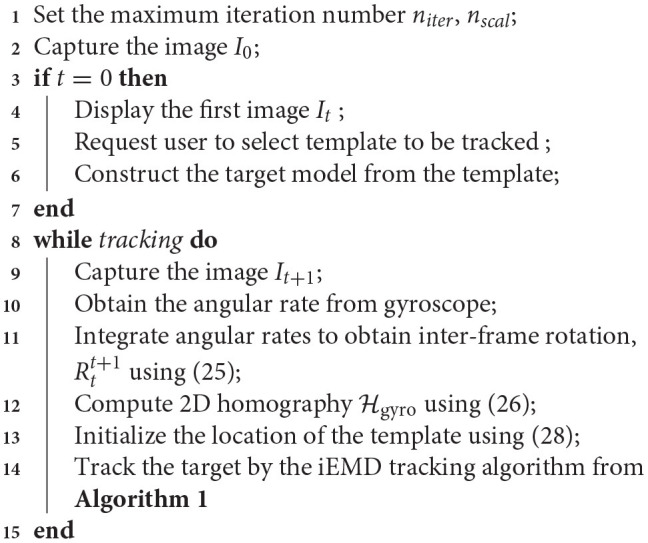
Gyro-aided iEMD tracking algorithm.

## 6. Experiments

In this section, the iEMD algorithm is validated on real datasets. The algorithm is implemented in MATLAB R2015b, the C code in Rubner et al. (2000) is adopted for the EMD calculation, and the software in Mairal et al. (2014) is used for sparse modeling. The platform is Microsoft Windows 7 professional with Intel(R) Core(TM) i5-4590 CPU. Eight publicly available datasets are chosen to validate the iEMD tracking algorithm. The main attributes of the video sequences are summarized in Table 1. The Car2, Walking, Woman, Subway, Bolt2, Car4, Human8, and Walking2 sequences are from the visual tracker benchmark (Wu et al., 2013) (CVPR 2013, http://www.visual-tracking.net). The length of the sequences varies between 128 and 913 frames with one object being tracked in each frame.

**Table 1:** The main attributes of the video sequences.

**Sequence**	**Frames**	**Image size**	**Target size**	**IV**	**SV**	**OCC**	**DEF**	**MB**	**FM**	**BC**
Car4	659	360 × 240	107 × 87	✓	✓					
Walking	412	768 × 576	24 × 79		✓	✓	✓			
Woman	550	352 × 288	21 × 95	✓	✓	✓	✓	✓	✓	
Subway	175	352 × 288	19 × 51			✓	✓			✓
Bolt2	293	480 × 270	34 × 64				✓			✓
Car2	913	320 × 240	64 × 52	✓	✓			✓	✓	✓
Human8	128	320 × 240	30 × 91	✓	✓		✓			
Walking2	500	384 × 288	31 × 115		✓	✓				

*Target size, the initial target size in the first frame; IV, illumination variation; SV, scale variation; OCC, occlusion; DEF, deformation; MB, motion blur; FM, fast motion; BC, background clutters*.

The tracker is initialized with the ground-truth bounding box of the target in the first frame. Then the tracking algorithm runs till the end of the sequence and generates a series of the tracked bounding boxes. Tracking results from consecutive frames are compared with the ground truth bounding boxes provided by this dataset. The relative overlap measure is used to evaluate this algorithm as (Wu et al., 2013)

(29) $$\begin{array}{l} \text{overlap}=\frac{R_{tr}\cap R_{gt}}{R_{tr}\cup R_{gt}} \end{array}$$

where **R**_*tr*_ is the tracking result, represented by the estimated image region occupied by the tracked object, **R**_*gt*_ is the ground truth bounding box. **R**_*tr*_∩**R**_*gt*_ is the intersection and **R**_*tr*_∪**R**_*gt*_ is the union of the two regions. The range of the relative overlap is from 0 to 100%.

### 6.1. Results for the iEMD tracker with sparse coding histograms

In this subsection, the performance of the iEMD tracker with sparse coding histograms and the template update method is evaluated using the eight sequences. In our approach, the object windows are re-sized to 32 × 32 pixels for all the sequences, except for the Walking sequence, in which the object windows are resized to 64 × 32 pixels due to the smaller object size. The local patches in each object window are sampled with the size 16 × 16 pixels with step size 8 in sequences like Car4, Walking and Car2. For other sequences, the local patches in each object window are sampled with the size 8 × 8 pixels with step size 4. In the case of the abrupt motions of the object, 4 more particles are generated by moving the template in the surrounding area of the initial object position. For each particle, the template is enlarged and shrunk by 2% in case of the scale variations. Video 1 shows the comparisons of tracking methods on eight tracking video sequences (Supplementary Material section).

**Figure 2:**
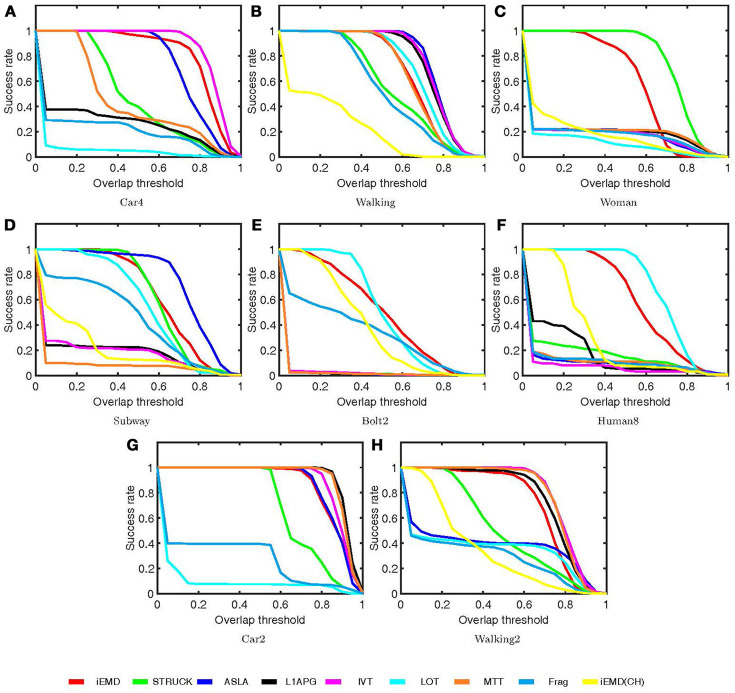
Success plots **(A–H)** for the nine tracking algorithms on the eight sequences.

The performance of the proposed algorithm is compared with eight state-of-the-art tracking algorithms on eight video sequences. These state-of-the-art trackers include: ASLA (Jia et al., 2012), Frag (Adam et al., 2006), IVT (Ross et al., 2008), L1APG (Mei and Ling, 2011), LOT (Oron et al., 2012), MTT (Zhang et al., 2012), STRUCK (Hare et al., 2011), and iEMD(CH) (Yao et al., 2016; Yao and Dani, 2017). The source codes of the trackers are downloaded from the corresponding web pages and the default parameters are used. The average percentage overlap obtained by all the tracking algorithms on eight video sequences are reported in Table 2. The iEMD tracker achieves the highest average overlap over all the sequences. The iEMD tracker also achieves the second best tracking results on the 5 out of 8 sequences. In Figure 2, the tracking results of the eight video sequences are shown. The success plot shows the ratios of frames at the different thresholds of the relative overlap values varied from 0 to 1.

**Table 2:** The average overlap (in percentage) obtained by the tracking algorithms on eight datasets.

**Sequence**	**ASLA**	**Frag**	**IVT**	**L1APG**	**LOT**	**MTT**	**STRUCK**	**iEMD(CH)**	**iEMD**
Car4	75.4	18.8	87.6	24.9	4.2	44.7	48.9	–	82.0
Walking	77.2	53.7	76.6	75.3	70.4	66.6	57.1	22.5	67.1
Woman	14.8	14.7	14.7	16.2	8.9	16.7	73.2	15.7	60.7
Subway	75.6	44.0	15.9	16.2	56.0	6.8	62.6	17.9	63.9
Bolt2	1.1	32.6	1.6	1.1	51.8	1.1	1.2	38.9	50.1
Car2	86.4	25.9	89.3	92.4	8.6	91.5	68.8	–	86.2
Human8	8.8	9.7	5.5	15.6	70.4	9.8	14.7	31.1	60.2
Walking2	37.1	27.4	79.5	75.6	33.5	78.5	51.0	34.5	71.2
Average	47.1	28.4	46.3	39.7	38.0	39.5	47.2	26.8	67.7

*For each sequence, the first, second, and third ranks are marked in red, green, and blue respectively. The last row is the average value of the percentage overlap for each tracker overall sequences*.

**Figure 3:**
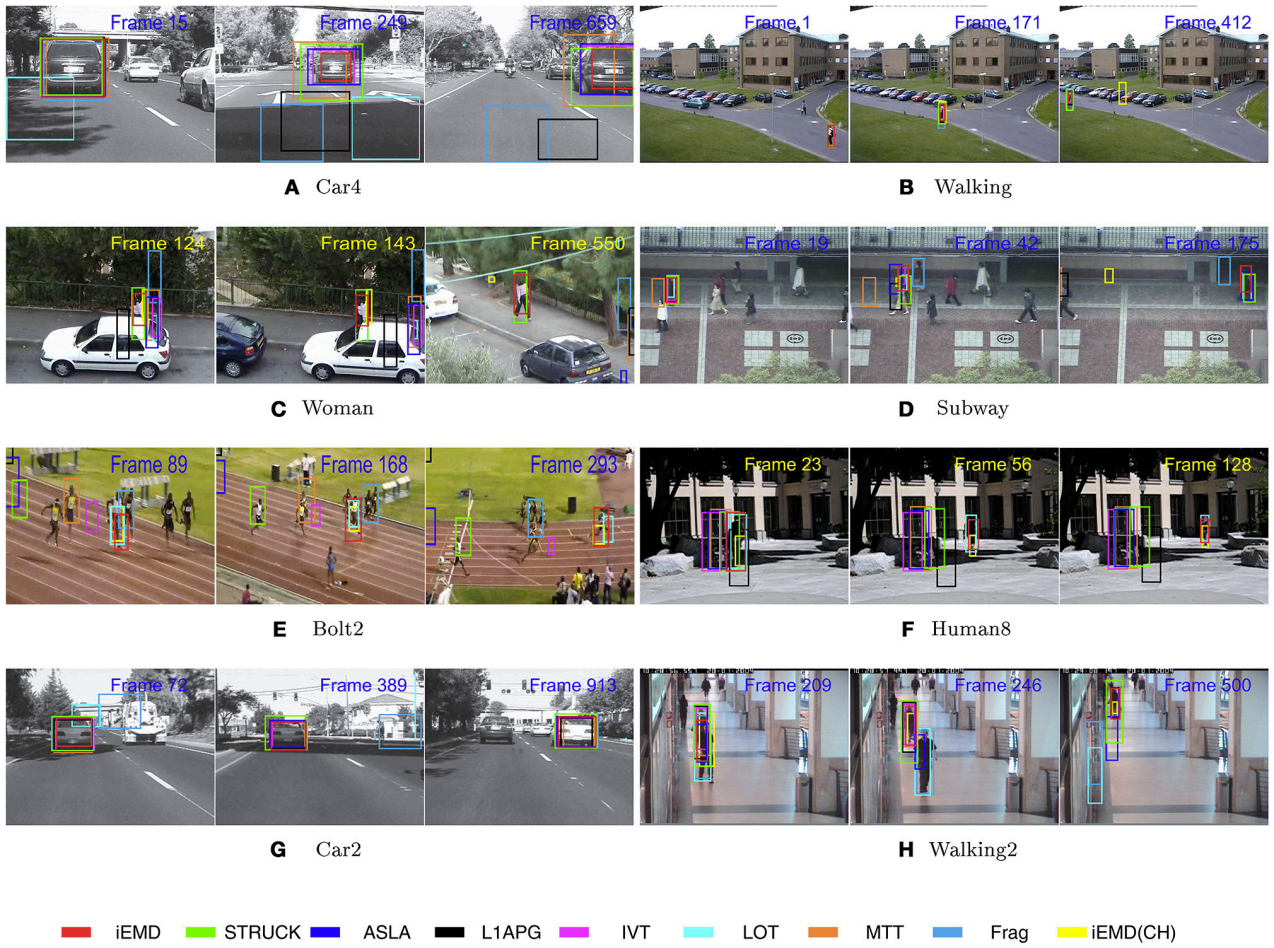
The visual tracking results obtained by the nine tracking algorithms on the eight video sequences (No permission is required from the copyright holders and/or the depicted individuals for the use of these images. The original images are obtained from the CVPR 2013 database: http://cvlab.hanyang.ac.kr/tracker_benchmark/datasets.html).

Representative tracking results obtained by iEMD algorithm are shown in Figure 3. In the Human8 and Bolt2 sequences, the targets have significant illumination variations, and deformations, respectively. Only LOT, iEMD and iEMD(CH) trackers are able to track targets in all the frames. The size of the template estimated by the iEMD(CH) tracker is shrinked and not accurate. The LOT and iEMD trackers use the EMD as the similarity measure and their appearance models are based on local image patches, which make the trackers more robust to illumination changes and deformations (Rubner et al., 2000; Oron et al., 2012). In woman sequence, all the trackers start to drift away from the target in frame 124 except for the iEMD and STRUCK trackers. For the Car2 and Car4 sequences, there are significant illumination changes when the targets pass underneath the trees and the overpasses. The LOT and Frag trackers start drifting away from frame 72 in Car2 sequences. In Car4 sequence, the LOT tracker starts to lose the target from frame 15, and the Frag and L1APG trackers drift away when the car passes the overpass in frame 249. In Walking2 sequence, the LOT, Frag, and ASLA trackers start tracking the wrong target in frame 246, due to the similar colors of the clothes between the two people.

### 6.2. Results for the gyro-aided iEMD tracking algorithm

The test of the gyro-aided iEMD tracking algorithm is conducted using the sequence including 100 frames from the dataset provided by CMU (Hwangbo et al., 2011). The images are taken in front of a desk with motions, such as shaking and rotation. The frame sequences have a resolution of 640 × 480 at 30 frames per second (FPS). The gyroscope is carefully aligned with the camera and the tri-axial gyroscopic values are sampled at 11Hz in the range of ±200deg/sec (Hwangbo et al., 2011). Using the time stamps of the camera and the gyroscope, the angular rate data are synchronized with the frames captured by the camera.

**Figure 4:**
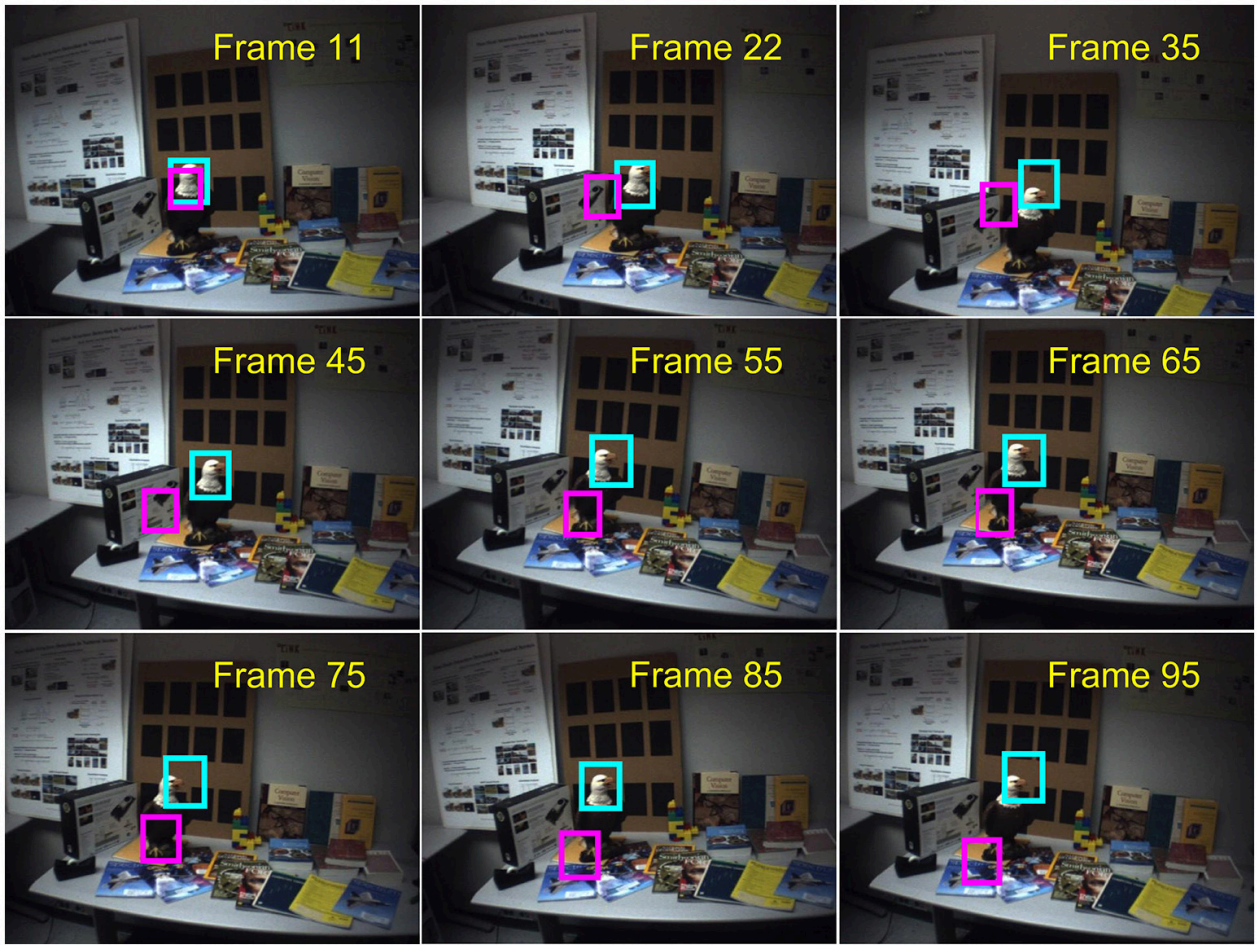
Results of the iEMD tracker in presence of rapid camera motion; the magenta boxes indicate the results of the iEMD tracker without the gyroscope information, and the cyan boxes indicate the results of the gyro-aided iEMD tracker (No permission is required from the copyright holders and/or the depicted individuals for the use of these images. The original images are obtained from the database: http://www.cs.cmu.edu/~myung/IMU_KLT/).

The comparisons between the tracking results using the iEMD tracker with and without the gyroscope information are illustrated in Figure 4. The head of the eagle is chosen as the target and the ground truth is manually labeled in each frame. The magenta box indicates the estimated image region without using the gyroscope data, and the cyan box is the tracking results of the gyro-aided iEMD tracker. Without the gyroscope data, the tracker loses the target after the frame 15. However, the head of the eagle is successfully tracked with our gyro-aided iEMD tracking algorithm.

The performances of the iEMD tracker with and without the gyroscope information on the CMU sequence are summarized in Table 3. The value of the average overlap, the percentage of the total frame numbers of which the overlap is greater than 0 and 40% are reported. Gyroscope information provides a good initial position for the iEMD tracker to estimate the location of the target. Thus, the gyro-aided iEMD tracking algorithm is robust to the rapid movements of the camera.

**Table 3:** Evaluation results on the CMU dataset using the iEMD tracker with and without the gyroscope information.

**Relative overlap with the ground truth**	**Gyro-aided**	**No gyro-aided**
Average overlap (%)	43.6	10.6
Overlap >0	100	13.3
Overlap >40%	58.9	1.1

## 7. Discussion

As a cross-bin metric for the comparison of the histograms, the advantages of the EMD are demonstrated in situations such as illumination variation, object deformation and partial occlusion. The iEMD algorithm uses the transportation-simplex method for calculating the EMD. The practical running time complexity of the transportation-simplex method is supercubic [a complexity in Ω(*N*^3^)⋂*O*(*N*^4^)] (Rubner et al., 2000), where *N* represents the number of the histogram bins. Other algorithms for calculating the EMD can be used to further reduce the running time (Ling and Okada, 2007; Pele and Werman, 2009). For our current impelementation in MATLAB, the average run time computed over the eight test sequences is 1.4 FPS. Compared to the algorithms used in Table 2, which has an average run time of 1 FPS or less (for algorithms implemented in MATLAB), iEMD algorithm performs better in terms of FPS (Wu et al., 2013). Furthermore, the experimental results, especially the Human8 and Bolt2 sequences, show that the iEMD tracker is robust to the appearance variations. The experimental results of Walking2 show that the iEMD tracker can discriminate the target from the surroundings with similar colors. The tracking results from Woman and Subway sequences demonstrate the robustness to partial occlusions. In Figures 2, 3 and Table 2, the tracking results of the iEMD(CH) (Yao et al., 2016; Yao and Dani, 2017) are also presented using six out of eight videos which has color images. The iEMD(CH) algorithm cannot be tested on Car2 and Car4 videos because they have gray images. Since the sparse coding histogram is used as the appearance model and the template update method is adopted to handle the appearance changes of the target, the performance of the iEMD with the sparse coding histogram is significantly better than our prior work using the iEMD(CH).

## 8. Conclusion and future work

This paper presents iEMD and gyro-aided iEMD visual tracking algorithms. The local sparse representation is used as the appearance model for the iEMD tracker. The maximum-alignment-pooling method is used for constructing a sparse coding histogram which reduces the computational complexity of the EMD optimization. The template update algorithm based on the EMD is also presented. The iEMD tracker is robust to variations in appearance of the target, deformations and partial occlusions. Experiments conducted on eight publicly available datasets show that the iEMD tracker is robust to the illumination changes, deformations and partial occlusions of the target. To validate the gyro-aided iEMD tracking algorithm, experimental results from the CMU dataset, which contains rapid camera motion are presented. Without the gyroscope measurements, the iEMD tracker fails on the CMU dataset. With the help of the gyroscope measurements, the iEMD algorithm is able to lock onto the target and track it successfully. The above experimental results show that the proposed iEMD tracking algorithm is robust to the appearance changes of the target as well as the ego-motion of the camera. As a gradient descent based dynamic model, the iEMD tracker, which provides good location prediction, can be further improved with more effective particle filters. The metrics used by sparse coding, such as the largest sum of the sparse coefficients or the smallest reconstruction error, can be combined with the EMD to make the tracker more discriminant. In future, an efficient impelementation of iEMD tracker in C/C++ will be pursued.

## Author contributions

GY: co-developed the algorithm and coded and validated the results. AD: co-developed the algorithm and verified the results.

### Conflict of interest statement

The authors declare that the research was conducted in the absence of any commercial or financial relationships that could be construed as a potential conflict of interest.

## Supplementary material

Video 1 Comparisons of Tracking Methods.
